# Manipulation of Promyelocytic Leukemia Protein Nuclear Bodies by Marek’s Disease Virus Encoded US3 Protein Kinase

**DOI:** 10.3390/microorganisms9040685

**Published:** 2021-03-26

**Authors:** Yifei Liao, Blanca Lupiani, Sanjay M. Reddy

**Affiliations:** Department of Veterinary Pathobiology, College of Veterinary Medicine & Biomedical Sciences, Texas A&M University, College Station, TX 77843, USA; liao.yifei@tamu.edu (Y.L.); blupiani@tamu.edu (B.L.)

**Keywords:** Marek’s disease virus, US3, protein kinase, PML, SP100

## Abstract

Promyelocytic leukemia protein nuclear bodies (PML-NBs) are dynamic nuclear structures, shown to be important for herpesvirus replication; however, their role in regulating Marek’s disease virus (MDV) infection has not been studied. MDV is an oncogenic alphaherpesvirus that causes lymphoproliferative disease in chickens. MDV encodes a US3 serine/threonine protein kinase that is important for MDV replication and gene expression. In this study, we studied the role of MDV US3 in regulating PML-NBs. Using an immunofluorescence assay, we found that MDV US3 disrupts PML and SP100 in a kinase dependent manner. In addition, treatment with MG-132 (a proteasome inhibitor) could partially restore the levels of PML and SP100, suggesting that a cellular proteasome dependent degradation pathway is involved in MDV US3 induced disruption of PML and SP100. These findings provide the first evidence for the interplay between MDV proteins and PML-NBs.

## 1. Introduction

Upon infection with viruses, the host utilizes various anti-viral mechanisms to limit virus replication and spread. In turn, viruses have evolved to evade host immunity and promote viral replication. Nuclear domain 10, also known as promyelocytic leukemia protein nuclear bodies (PML-NBs), has been characterized as a host factor that restricts virus infection [1]. PML-NBs are dynamic nuclear macromolecular structures that consist of proteins, such as PML, SP100, Daxx, and SUMO-1 (small ubiquitin-related modifier 1), and other transitory factors [2]. PML-NBs have been shown to be involved in a variety of cellular activities, including transcription, apoptosis, DNA damage repair, and intrinsic immunity [3,4,5,6].

Increasing evidence suggests that PML-NBs are tightly associated with herpesvirus genome replication and gene transcription [1]. It has been reported that herpes simplex virus type 1 (HSV-1) encoded ICP0 protein contains an E3 ubiquitin ligase activity domain that could mediate the degradation of PML and SP100, and that this process is important for HSV-1 lytic replication and reactivation [7,8]; human cytomegalovirus (HCMV) encoded immediate early protein 1 (IE1) and IE2 have been shown to colocalize with and disrupt the PML-NBs, allowing for the efficient lytic replication of HCMV [9]; Epstein–Barr virus (EBV) encoded BZLF-1, a viral bZIP protein, has been reported to regulate virus replication by disrupting PML-NBs [10]; K-bZIP (also known as K8), encoded by Kaposi’s sarcoma-associated herpesvirus (KSHV), localizes to PML-NBs to establish virus replication compartments [11,12], and PML has been identified as a positive factor for KSHV lytic replication [13]. In addition, the HSV-2 encoded US3 serine/threonine protein kinase, a conserved viral protein kinase within the *Alphaherpesvirinae* subfamily, has been shown to disrupt PML-NBs in a kinase activity dependent manner [14].

However, the regulation of PML-NBs by Marek’s disease virus (MDV) has not been studied. MDV, a member of the *Mardivirus* genus also known as *Gallid alphaherpesvirus* 2 (GaHV-2), is an oncogenic and highly contagious avian alphaherpesvirus that causes T cell lymphomas in chickens [15,16]. The MDV genome is ~177 kb and encodes more than 100 putative functional proteins [15]. Similar to other alphaherpesviruses, MDV encodes a US3 serine/threonine protein kinase, which is a multi-functional protein involved in virus replication, morphogenesis, and pathogenesis, as well as apoptosis inhibition and transcriptional regulation [17,18,19,20].

## 2. Materials and Methods

### 2.1. Cells, Plasmids and Chemicals

293 cells were maintained in Dulbecco’s modified Eagle medium (DMEM) (Thermo Fisher Scientific, Waltham, MA, USA) supplemented with 10% fetal bovine serum at 37 °C in the presence of 5% CO_2_, and were used for transfection experiments.

pcDNA mammalian expression vectors of N-terminal FLAG tagged wild type US3 (MDV strain 686) [21] and kinase dead US3 (US3-K220A) were described previously [17,18]. N-terminal HA tagged human SP100 (NCBI Reference Sequence: NM_001080391.2) was amplified from human cDNA and cloned into pcDNA mammalian expression vector using standard protocols.

MG-132 was reconstituted with DMSO to 10 mM stock and used at 10 µM.

### 2.2. Immunofluorescence Assay and Cells Counting

293 cells seeded on coverslips were transfected with pcDNA expression plasmids using polyethylenimine (PEI, 1 mg/mL) reagent. Forty-eight hours later, cells were fixed and permeabilized with 3.7% formaldehyde, 1.0% Triton X-100, and 1.0% NP-40, sequentially. For MG-132 and DMSO treatment, twenty-four hours after transfection, cells were treated with DMSO or MG-132 (10 µM) overnight, fixed and permeabilized, as stated above. Cells were blocked in 5% nonfat milk (in PBS) for 1 hour, followed by incubation with primary antibodies (mouse anti-PML antibody (Santa Cruz Biotechnology, #sc-377390, Dallas, TX, USA); mouse anti-HA antibody (Invitrogen, #26183, Carlsbad, CA, USA); rabbit anti-FLAG antibody (Invitrogen, #740001)) and corresponding secondary antibodies (Texas Red conjugated goat anti-rabbit antibody (Invitrogen, #T-2767); Alexa flour 488 conjugated goat anti-mouse antibody (Invitrogen, #A-11001); Texas Red conjugated goat anti-mouse antibody (Invitrogen, #T-862); Alexa flour 488 conjugated goat anti-rabbit antibody (Invitrogen, #A-11008)) for 1 h each at room temperature. Cell nuclei were stained with 4′, 6-diamidino-2-phenylindole (DAPI). Coverslips were then mounted on glass slides using ProLong Diamond Antifade Mountant, and cells were visualized using a Zeiss LSM 780 NLO multiphoton microscope.

For each group, percentage of cells positive for PML or SP100, and the number of PML or SP100 dots per PML or SP100 positive cell were counted from 50 randomly selected US3 or US3-K220A positive cells using Image J software. Only cells that were positive for PML or SP100 were used to calculate the average number of PML or SP100 dots per cell.

### 2.3. Statistical Analysis

Cell and dot counting results are presented as the average number from two independent transfection experiments, with error bars representing standard deviation (SD). Student’s t test was performed between indicated groups and statistical significance is indicated with an asterisk. *: *p* < 0.05; **: *p* < 0.01.

## 3. Results and Discussion

**Figure 1:**
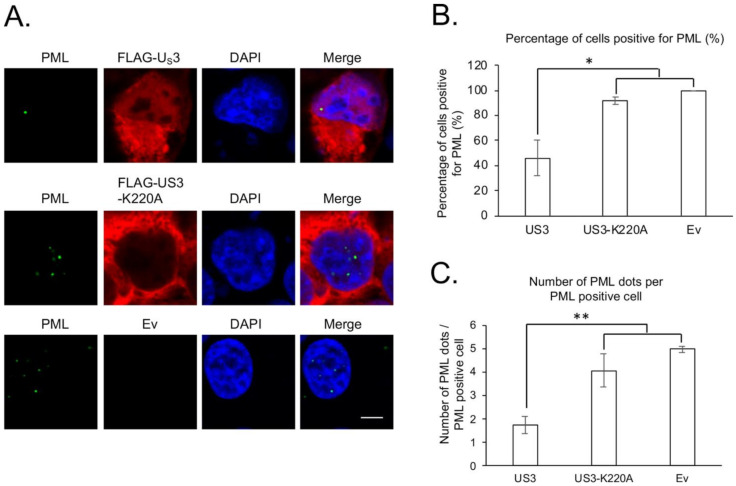
Effect of Marek’s disease virus (MDV) U_S_3 on promyelocytic leukemia (PML) protein. (**A**) 293 cells were transfected with pcDNA expressing FLAG-U_S_3, FLAG-U_S_3-K220A (kinase dead), or empty vector (Ev). Forty-eight hours later, cells were fixed and permeabilized for immunofluorescence assay using primary rabbit anti-FLAG antibody and mouse anti-PML antibody, followed by secondary Texas Red conjugated goat anti-rabbit antibody and Alexa flour 488 conjugated goat anti-mouse antibody. 4′, 6-diamidino-2-phenylindole (DAPI) was used to stain cell nuclei. All images were taken with a confocal microscope at the same magnification, and representative images are presented. Scale bar = 10 µm. (**B**,**C**) For each group, the percentage of cells positive for PML (**B**) and the number of PML dots per PML positive cell (**C**) were counted from 50 randomly selected U_S_3 or U_S_3-K220A transfection positive cells using Image J software. Only cells that were positive for PML were used to calculate the average number of PML dots per cell. Results are presented as average number from two independent transfection experiments; error bars represent standard deviation (SD). Student’s *t* test was performed between U_S_3, or U_S_3-K220A and Ev transfected cells, and statistical significance is indicated with an asterisk. *: *p* < 0.05; **: *p* < 0.01.

**Figure 2:**
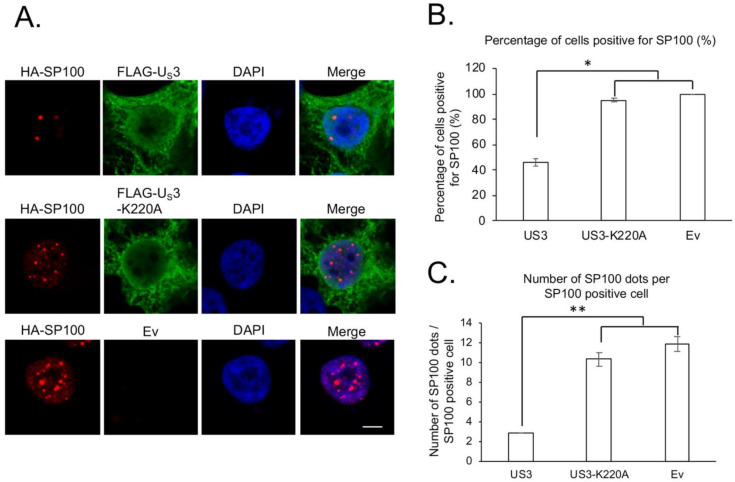
Effect of MDV U_S_3 on SP100 protein. (**A**) pcDNA expressing HA-SP100 was co-transfected with FLAG-U_S_3, FLAG-U_S_3-K220A (kinase dead), or empty vector (Ev) into 293 cells. Forty-eight hours later, cells were fixed and permeabilized for immunofluorescence assay using primary mouse anti-HA antibody and rabbit anti-FLAG antibody, followed by secondary Texas Red conjugated goat anti-mouse antibody and Alexa flour 488 conjugated goat anti-rabbit antibody. DAPI was used to stain cell nuclei. All images were taken with a confocal microscope at the same magnification, and representative images are presented. Scale bar = 10 µm. (**B**,**C**) For each group, the percentage of cells positive for HA-SP100 (**B**) and the number of HA-SP100 dots per HA-SP100 positive cell (**C**) were counted from 50 randomly selected U_S_3 or U_S_3-K220A transfection positive cells using Image J software. Only cells that were positive for HA-SP100 were used to calculate the average number of HA-SP100 dots per cell. Results are presented as the average number from two independent transfection experiments; error bars represent standard deviation (SD). Student’s *t* test was performed between U_S_3 or U_S_3-K220A and Ev transfected cells, and statistical significance is indicated with an asterisk. *: *p* < 0.05; **: *p* < 0.01.

To better understand the interplay between MDV proteins and PML-NBs, we examined the role of US3 from a very virulent plus MDV strain (686) in regulating the distribution and levels of PML-NBs components, PML and SP100. Our results show that the transfection of wild type US3 or kinase dead US3 (US3-K220A) did not affect the subcellular distribution of PML (Figure 1A) and SP100 (Figure 2A). However, expression of US3, but not US3-K220A, resulted in a significant reduction in the number of PML (Figure 1B and Table S1) and SP100 (Figure 2B and Table S2) positive cells, as well as the number of PML (Figure 1C and Table S1) and SP100 (Figure 2C and Table S2) dots per cell. Taken together, these results suggest that MDV US3 exhibits an activity similar to HSV-2 US3 and disrupts PML-NBs in a kinase activity dependent manner.

**Figure 3:**
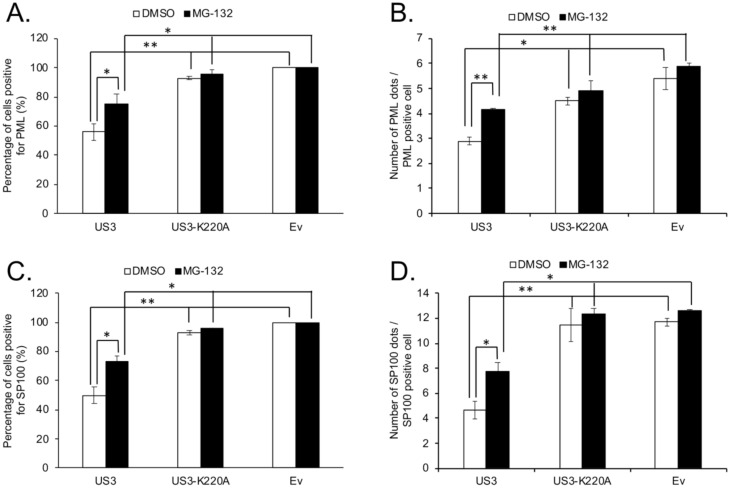
Effect of MG-132 treatment on MDV U_S_3 induced disruption of PML and SP100. (**A**,**B**) 293 cells were transfected with plasmid expressing FLAG-U_S_3, FLAG-U_S_3-K220A (kinase dead), or empty vector (Ev). Twenty-four hours later, cells were treated with DMSO or MG-132 (10 µM) overnight. The next day, cells were fixed and permeabilized for immunofluorescence assay. For each group, the percentage of cells positive for PML (**A**) and the number of PML dots per PML positive cell (**B**) were counted from 50 randomly selected U_S_3 or U_S_3-K220A transfection positive cells using Image J software. Only cells that were positive for PML were used to calculate the average number of PML dots per cell. (**C**,**D**) pcDNA expressing HA-SP100 was co-transfected with FLAG-U_S_3, FLAG-U_S_3-K220A, or Ev into 293 cells. Twenty-four hours later, cells were treated with DMSO or MG-132 (10 µM) overnight. The next day, cells were fixed and permeabilized for immunofluorescence assay. For each group, the percentage of cells positive for HA-SP100 (**C**) and the number of HA-SP100 dots per HA-SP100 positive cell (**D**) were counted from 50 randomly selected U_S_3 or U_S_3-K220A transfection positive cells using Image J software. Only cells that were positive for HA-SP100 were used to calculate the average number of HA-SP100 dots per cell. All results are presented as average number from two independent transfection experiments; error bars represent standard deviation (SD). Student *t* test was performed between indicated groups, and statistical significance is indicated with an asterisk. *: *p* < 0.05; **: *p* < 0.01.

**Figure 4:**
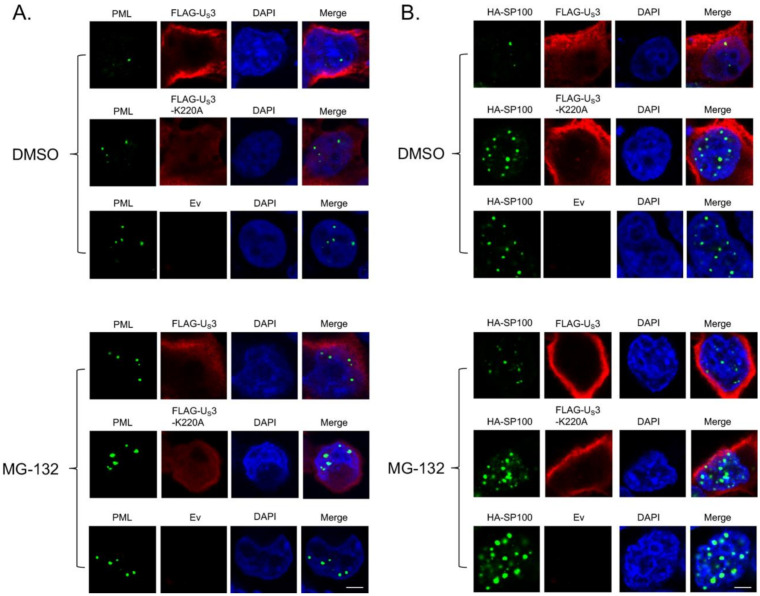
Effect of MG-132 treatment on MDV U_S_3 induced disruption of PML and SP100. (**A**) 293 cells were transfected with pcDNA expressing FLAG-U_S_3, FLAG-U_S_3-K220A (kinase dead), or empty vector (Ev). Twenty-four hours later, cells were treated with DMSO or MG-132 (10 µM) overnight. The next day, cells were fixed and permeabilized for immunofluorescence assay using primary mouse anti-PML antibody and rabbit anti-FLAG antibody, followed by secondary Alexa flour 488 conjugated goat anti-mouse antibody and Texas Red conjugated goat anti-rabbit antibody. (**B**) pcDNA expressing HA-SP100 was co-transfected with FLAG-U_S_3, FLAG-U_S_3-K220A, or Ev into 293 cells. Twenty-four hours later, cells were treated with DMSO or MG-132 (10 µM) overnight. The next day, cells were fixed and permeabilized for immunofluorescence assay using primary mouse anti-HA antibody and rabbit anti-FLAG antibody, followed by secondary Alexa flour 488 conjugated goat anti-mouse antibody and Texas Red conjugated goat anti-rabbit antibody. DAPI was used to stain cell nuclei. All images were taken with a confocal microscope at the same magnification and representative images are presented. Scale bar = 10 µm.

It was shown that treatment with MG-132, a proteasome inhibitor, could partially rescue the number of PML dots disrupted by HSV-2 US3, suggesting the involvement of the proteasome degradation pathway in this process [14]. We next examined the inhibitory effect of MG-132 treatment on MDV US3 mediated disruption of PML and SP100. Our results show that, compared to DMSO (control), MG-132 treatment significantly enhanced the number of cells positive for PML (Figure 3A and Table S3) and SP100 (Figure 3C and Table S4) in US3 transfected cells, but it had no effect on US3-K220A or Ev transfected cells. In addition, we observed that, after MG-132 treatment, the number of cells positive for PML (Figure 3A and Table S3) and SP100 (Figure 3C and Table S4) in US3 transfected cells was still significantly less than in Ev transfected cells, suggesting that the treatment of MG-132 could only partially rescue the elimination of PML and SP100 mediated by US3, which is consistent with a previous report on HSV-2 US3 [14]. Similar patterns applied to the effect of US3 on the number of PML and SP100 dots per cell, and MG-132 treatment partially restored the number of PML (Figure 3B and Figure 4A, Table S3) and SP100 (Figure 3D and Figure 4B, Table S4) dots in cells that were disrupted by the expression of US3.

Recently, we reported that MDV US3 phosphorylates chicken CREB to regulate cellular and viral gene expression and phosphorylates chicken histone deacetylase 1 and 2 (HDAC1 and 2) to regulate virus replication and pathogenesis [17,18]. Here, we demonstrated that MDV US3 disrupts PML-NBs, including PML and SP100, in a kinase activity and proteasome dependent manner, further expanding our knowledge about the functions of MDV US3. Although the SP100 ortholog has not been identified in the chicken genome, chicken PML ortholog exhibits a 50% similarity to human PML and has been shown to form nuclear dots similar to human PML [22], indicating that MDV US3 may be able to disrupt chicken PML in a manner similar to human PML. Considering the importance of PML-NBs in herpesvirus replication, it is possible that MDV US3 facilitates the replication of MDV by disrupting PML-NBs. Since, MDV US3 exhibits functions conserved among homologs encoded by other alphaherpesviruses, our study provides valuable data for future research towards a better understanding of the functions of alphaherpesvirus US3 protein kinases. We tried to confirm our results in chicken cells, such as primary embryonic fibroblasts (CEF) and DF-1, an embryonic fibroblast cell line, but the commercial PML antibodies did not cross-react with chicken PML. Future studies will focus on custom chicken PML antibody production and on verifying the results in chicken cells, as well as the elucidation of the biological importance of MDV US3 induced disruption of PML-NBs.

## Data Availability

Data is contained within the article or supplementary material.

## Supplementary Materials

The following are available online at https://www.mdpi.com/article/10.3390/microorganisms9040685/s1.
