# Supplementary material for: Manipulation of Promyelocytic Leukemia Protein Nuclear Bodies by Marek’s Disease Virus Encoded US3 Protein Kinase
Source: Microorganisms. 2021 Mar 26;9(4):685. doi: 10.3390/microorganisms9040685 (PMC8066686; doi:10.3390/microorganisms9040685)
Supplement: Supplementary file 1 [file microorganisms-09-00685-s001.pdf]

Table S1. Effect of MDV U_S3 on PML expression.

	Cells positive for PML	Percentages of cells positive for PML (%)	Numbers of PML dots/ PML positive cell
U_S3	23 ± 7.1 *	46 ± 14.2 *	1.75 ± 0.4 **
U_S3-K220A	46 ± 1.4	92 ± 2.8	4.1 ± 0.7
Ev	50 ± 0	100 ± 0	5 ± 0.1

Values are presented as average ± standard error (SD). Student t test was performed between US3, or US3-K220A and empty vector (Ev) transfected cells, and statistical significance is indicated with asterisk. *: $p < 0.05$; **: $p < 0.01$.

Table S2. Effect of MDV U_S3 on SP100 expression.

	Cells positive for SP100	Percentage of cells positive for SP100 (%)	Numbers of SP100 dots / SP100 positive cell
U_S3	23 ± 1.4 *	46 ± 2.8 *	2.9 ± 0 **
U_S3-K220A	47.5 ± 0.7	95 ± 1.4	10.4 ± 0.6
Ev	50 ± 0	100 ± 0	11.9 ± 0.7

Values are presented as average ± standard error (SD). Student t test was performed between US3, or US3-K220A and empty vector (Ev) transfected cells, and statistical significance is indicated with asterisk. *: $p < 0.05$; **: $p < 0.01$.

Table S3. Effect of MG-132 treatment on MDV U_S3 induced disruption of PML.

		Percentage of cells positive for PML (%)	Numbers of PML dots/ PML positive cell
U_S3	DMSO	56 ± 5.6	2.9 ± 0.1
	MG-132	75 ± 7	4.15 ± 0.1
U_S3-K220A	DMSO	93 ± 1.4	4.5 ± 0.1
	MG-132	96 ± 2.8	4.9 ± 0.4
Ev	DMSO	100 ± 0	5.4 ± 0.4
	MG-132	100 ± 0	5.9 ± 0.1

Values are presented as average ± standard error (SD).

Table S4. Effect of MG-132 treatment on MDV U_S3 induced disruption of SP100.

		Percentage of cells positive for SP100 (%)	Number of SP100 dots/ SP100 positive cell
U_S3	DMSO	50 ± 5.6	4.7 ± 0.7
	MG-132	73 ± 4.2	7.8 ± 0.7
U_S3-K220A	DMSO	93 ± 1.4	11.45 ± 1.3
	MG-132	96 ± 0	12.4 ± 0.4
Ev	DMSO	100 ± 0	11.7 ± 0.3
	MG-132	100 ± 0	12.6 ± 0.1

Values are presented as average ± standard error (SD).
